# Shiraz Guideline for the Management of Patients with Brain Death

**Published:** 2014-02-01

**Authors:** M. Firoozifar, M. B. Khosravi, S. Ghafari, M. A. Sahmeddini, H. Eghbal, H. Salahi, A. Bahador, S. Nikeghbalian, K. Kazemi, A. R. Shamsaeefar, S. Gholami, E. Farhadi, M. A. Shahnazi, S. A. Malek-Hosseini

**Affiliations:** *Shiraz Organ Transplant Center, Namazi Hospital, Shiraz, Iran*

## INTRODUCTION

The first kidney, liver and pancreas transplantations in Iran were carried out in Shiraz University of Medical Sciences in 1968, 1992 and 2006, respectively. In the course of time, the need for transplanting organs from brain dead cases has increased in a way that transplantation section of the University is currently practicing more than 400 liver, 40 pancreas and 250 kidney transplantations annually. The fact that a large number of patients waiting in the list for transplantation expire before receiving organs reflects the significance of the process of the managing and maintaining brain dead cases. Proper implementation of maintenance procedures in cases of brain death increases the chance of successful transplantation.

Preparing a guideline for managing brain dead cases can serve as a great step toward standardization of the process of providing proper medical services for brain dead cases and as a result for receivers of organs. Accordingly, the first guideline for managing brain dead cases in Iran has been developed in Shiraz University of Medical Sciences and can be applied in other centers. In the course of preparing this guideline, we precisely reviewed the latest guidelines presented by pioneer countries in this domain such as Spain, the USA, the UK, Australia and Belgium, and used precious experiences of team of specialists of anesthesia in organ transplant center of Shiraz University of Medical Sciences in order to make the guideline sound more native.

General Purposes

The purpose of this guideline is to assess the principles of maintaining brain dead cases after diagnosis in the ICU until organ removal in the surgery.

The most important medical interventions practiced in brain dead cases include:

1. Respiratory aids and ventilator settings.
2. Hemodynamic interventions.
3. Interventions to control body fluids and electrolytes.
4. Cardiovascular aids.
5. Hormonal treatments.
6. Regulation of the body temperature.
7. Monitoring.

Specific Purposes

 Respiratory aids and ventilator settings: 

In order to avoid atelectasis and hypoxia in brain dead cases, the airways have to be kept hygienic. Therefore, it is recommended that the employed respiratory tube should be of its maximum size suitable for the patient based on his/her age and gender, the airways be regularly suctioned, and both side of the patient’s lungs be examined by a stethoscope after each suctioning or tube exchange. In brain dead cases, hypoxia must be diagnosed and treated immediately.

The proposed instructions and purposes in this section:

An *O*_2_* Sat* of 95%–100% is acceptable. *FIo*_2_ could be raised to 100%. Note that if the patient’s lungs are supposed to be transplanted, the minimum *FIo*_2_ that can raise the patient’s saturation to 98% is acceptable. A *PaO*_2_ of 90–100 mm Hg, a *Pco*_2_ of 35–45 mm Hg, and a *pH* of 7.35–7.45 are acceptable. Note that apnea test done for the diagnosis of brain death may lead to a considerable increase in *Pco*_2_ and respiratory acidosis. Therefore, special care is required during the test and after that to avoid respiratory acidosis, which may be realized through successive arterial blood gas analyses and a change in ventilator’s status. If *pH* falls below 7.25 and at the same time no respiratory acidosis is observed (*Pco*_2_ is normal), the metabolic acidosis should be treated by administration of sodium bicarbonate according to the following formula:

The volume of sodium bicarbonate= 0.3 × body weight (kg) × base excess (BE)

For each brain dead patient it is preferable to apply PEEP up to 5 cm H_2_O. The recommended value for the tidal volume is 8–10 mL/kg at most. The peak air way pressure should be kept below 30 mm Hg, especially if the patient’s lungs are supposed to be transplanted.

Hemodynamic interventions: 

Hypotension is a common problem among brain dead patients and must be diagnosed and cured immediately. Even short periods of low blood pressure in these cases may have harmful effects on the organs.

The proposed instructions and purposes in this section:

The mean arterial pressure (MAP) should be ≥60 mm Hg. Note that in patients aged over 60 year, the minimum MAP is set equal to the patient’s age; for example, for a 65-year-old patient, the MAP should be ≥65 mm Hg. Systolic blood pressure must be constantly ≥100 mm Hg. A heart rate of 70-100 beats/min is acceptable. The minimum acceptable central venous pressure (CVP) is 12 mm Hg. If CVP reaches 12 mm Hg but MAP is less than 60 mm Hg, administration of inotropic drugs, such as dopamine, is necessary. If the patient’s age is over 60 year, a CVP up to 15 mm Hg is also acceptable. Pulmonary capillary wedge pressure and systemic vascular resistance are necessary to be measured, if the patient’s lungs are supposed to be transplanted. High blood pressure is not common among brain dead cases, though during the first stages of the brain death, systolic blood pressure of higher than 180 mm Hg might be observed which is due to brainstem involvement and usually turns to normal value spontaneously. If the systolic blood pressure is ≥180 mm Hg or the diastolic blood pressure is ≥120 mm Hg, or MAP is ≥140 mm Hg, the pressure should be lowered by infusion of sodium nitroprusside; in such cases, a physician should also be consulted.

Interventions to control body fluids and electrolytes 

In brain dead patients, “polyuria” is defined as a situation in which the volume of urine exceeds 500 mL/h. The most common cause of polyuria in brain dead patients is diabetes insipidus (DI) which may lead to increased sodium and plasma osmolarity and decreased potassium (for more information see: Hormonal treatments). In general, it is recommended to keep the urine volume of a brain dead patient between 1 and 3 mL/kg/h.

The proposed instructions and purposes in this section:

The recommended serum sodium level is <150 mEq/dL. If the patients’ blood sodium is higher, the administered fluids should be substituted by half-saline; if it exceeds 165 mEq/dL, then DW 5% should be added and at the same time, urine sodium and osmolarity should be tested for the probable diagnosis of DI. The recommended serum potassium level is between 3.5 and 5.5 mEq/dL. It is recommended that fluid therapy in brain dead cases begins with a Ringer’s solution or normal saline at a dose of 2 mL/kg/h. If CVP is <12 cm H_2_O, however, Gelofusion should be added to the administered fluids. Administration of albumin is recommended when a the serum albumin level is <3.5 g/dL. Hematocrit has to be ≥30%. The recommended hemoglobin level is above 10 g/dL.

Cardiovascular aids in brain dead cases 

In case of decreased blood pressure despite of sufficient fluid therapy, it is advisable to administer cardiovascular inotropic drugs.

The proposed instructions and purposes in this section:

The first drug of choice is dopamine, which has to be administered by infusion in doses of 10 µg/kg/min. The second choice is norepinephrine at a dosage determined by a physician. If a patient is suffering from congestive heart failure (CHF), dopamine should be substituted by epinephrine and a physician should decide on its dosage.

Hormonal treatments 

To attain the hemodynamic stability of brain dead patients, various hormonal deficiencies (*eg*, levothyroxine, steroid, insulin deficiencies) must be taken into consideration.

The proposed instructions and purposes in this section

Levothyroxine should be administered as 6 pills of 0.1 mg through a gastric tube. Every 12 hours, 15 mg/kg of methylprednisolone should be administered by the CVP line. Regular insulin should be infused at a dose of one unit per hour from the very beginning; the patient’s glucose level should be tested every hour and adjusted according to the Table 1.

**Table 1:** The recommended insulin dosage according to the patient’s glucose level

**Blood sugar levels (mg/dL)**	**Insulin doses (Units/h)**
<140	0
140–169	2
170–199	3
200–249	4
250–299	6
300–399	8
≥400	10

It is recommended that 2-4 µg of desmopressin being sprayed nasally for treating DI. Nephrologist consultation is recommended if a patient is diagnosed with DI.

Temperature regulation of the body 

Dead-brain cases suffer from an uncontrolled central body temperature (poikilothermia) which may have abnormal influences on vital organs such as heart and liver. Therefore, it is advisable to keep the body warm through forced air so that the central temperature is kept above 35 °C.

Monitoring brain dead cases 

Each patient with brain death should have a CVP, arterial line, ECG, temperature, pulse Oximeter, and capnograghy monitoring. Pulmonary artery catheterization is only necessary in cases of lung transplantation.

All brain dead cases must undergo CXR and ECG as soon as possible. In cases of heart transplantation, echocardiography, coronary angiography and consultation are recommended. Bronchoscopy is recommended in lung transplantations.

CBC, LFT, BUN, electrolytes, Cr, INR, PTT and PT tests should be requested for all patients with brain death.

This guideline has gone through final validation in a joint meeting attended by specialists of the Anesthesiology Section of the Transplantation Sector and the provisional team of transplantation sector of Shiraz University of Medical Sciences.
